# Precision in Total Knee Replacement: A Technical Note on the VELYS Robotic-Assisted Tibial-Femoral Approach

**DOI:** 10.7759/cureus.73104

**Published:** 2024-11-06

**Authors:** Ricky Edwin P Hutapea, Karina Sylvana Gani, Muhammad Budimansyah, Mitchel Mitchel, Carlina Surya, Erica Kholinne

**Affiliations:** 1 Orthopaedics and Traumatology, Gatam Institute, Eka Hospital, Tangerang, IDN; 2 General Practice, Gatam Institute, Eka Hospital, Tangerang, IDN; 3 Orthopaedics and Traumatology, Persahabatan Hospital, Jakarta, IDN; 4 Orthopaedics and Traumatology, Faculty of Medicine, Universitas Trisakti, Jakarta, IDN

**Keywords:** osteoarthritis, robotic assisted, robotic knee surgery, total knee replacement, velys

## Abstract

The VELYS robotic-assisted system (DePuy Synthes, Warsaw, IN, USA) in total knee replacement (TKR) enables precise bone registration and resection, reducing manual errors and minimizing soft tissue injury compared to conventional methods. This method will provide a more accurate implant position with a better postoperative range of motion (ROM). This technical note describes the steps in performing robotic-assisted TKR using the VELYS with a tibial-femoral approach. In more detail, this article will discuss surgical exposure and creating bone checkpoints and landmark acquisitions, tibial-femoral resection, and implant placement. Despite the existing benefits, the long-term advantages still need further investigation.

## Introduction

Total knee replacement (TKR) is one of the most commonly performed procedures, with over 100,000 cases annually in the United Kingdom [1]. The rising number of TKR, driven by increased awareness and high patient satisfaction, underscores the need for precision in bony resection, implant positioning, and ligamentous balancing. This is particularly crucial as the average patient age decreases and functional expectations and demands on implants increase, ensuring patient satisfaction and implant longevity [2]. Recent advances in surgical technology, including robotic surgery, have led to an increase in the use of personalized alignment strategies during TKR [3]. This technical note aims to describe the steps in performing robotic-assisted TKR with a tibial-femoral approach.

## Technical report

Robotic device and patient positioning

**Figure 1:**
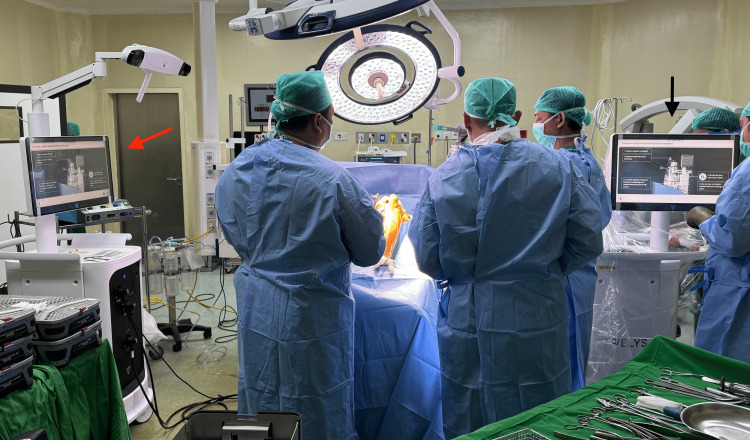
Operation room setup: (red arrow) satellite station and (black arrow) base station.

**Figure 2:**
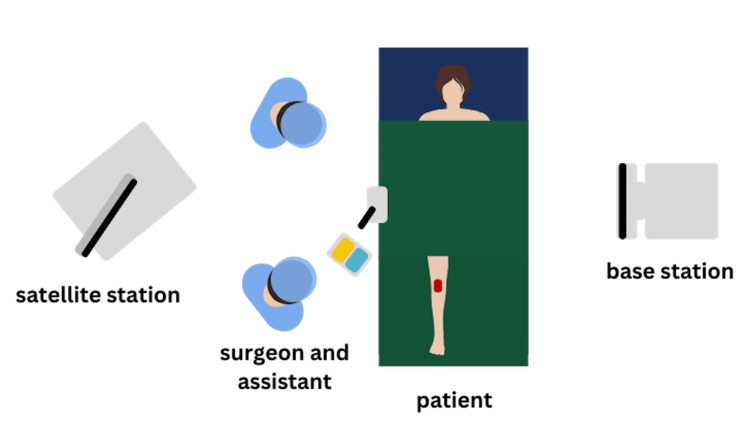
Illustration showing satellite station, base station, and patient positioning.

The VELYS robotic-assisted solution (DePuy Synthes, Warsaw, IN, USA) comprises a system with two stations: base and satellite [4]. The patient is positioned supine on the operating table with the knee flexed to approximately 90 degrees and supported by a padded footrest to ensure proper knee position and alignment (Figure 1 and Figure 2).

Surgical exposure, creation of bone checkpoints, and bone array setup

A medial parapatellar incision was made, and osteophytes were removed from the surgical site before the surgeon created a checkpoint in the tibial and femoral bone with the sharp tip of the array drill to make a slight indentation to place an array properly. The checkpoint should be distinct, clean from the soft tissue, and easily located.

**Figure 3:**
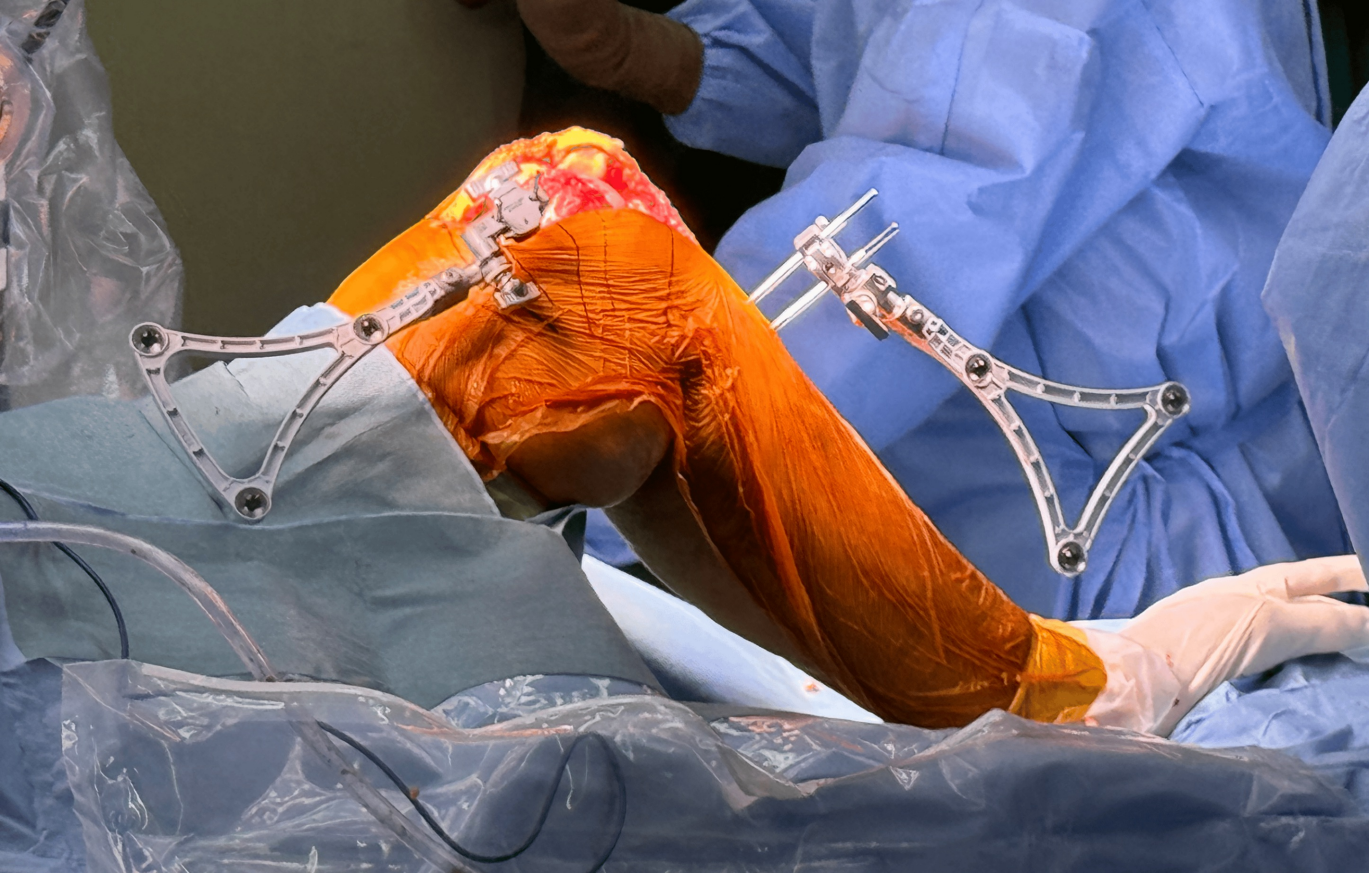
Bone array placement in the tibia and femur.

The femur array (convex) and tibia array (concave) are fixed reference points to create a "framing" of the system. The tibia array has to be placed 3-4 fingers below the incision and medial to the tibial crest. The femur array is inserted proximal-anteriorly to the medial epicondyle (Figure 3). The arrays have to be pointing toward the camera at all times.

Bone checkpoints and landmark acquisition

**Figure 4:**
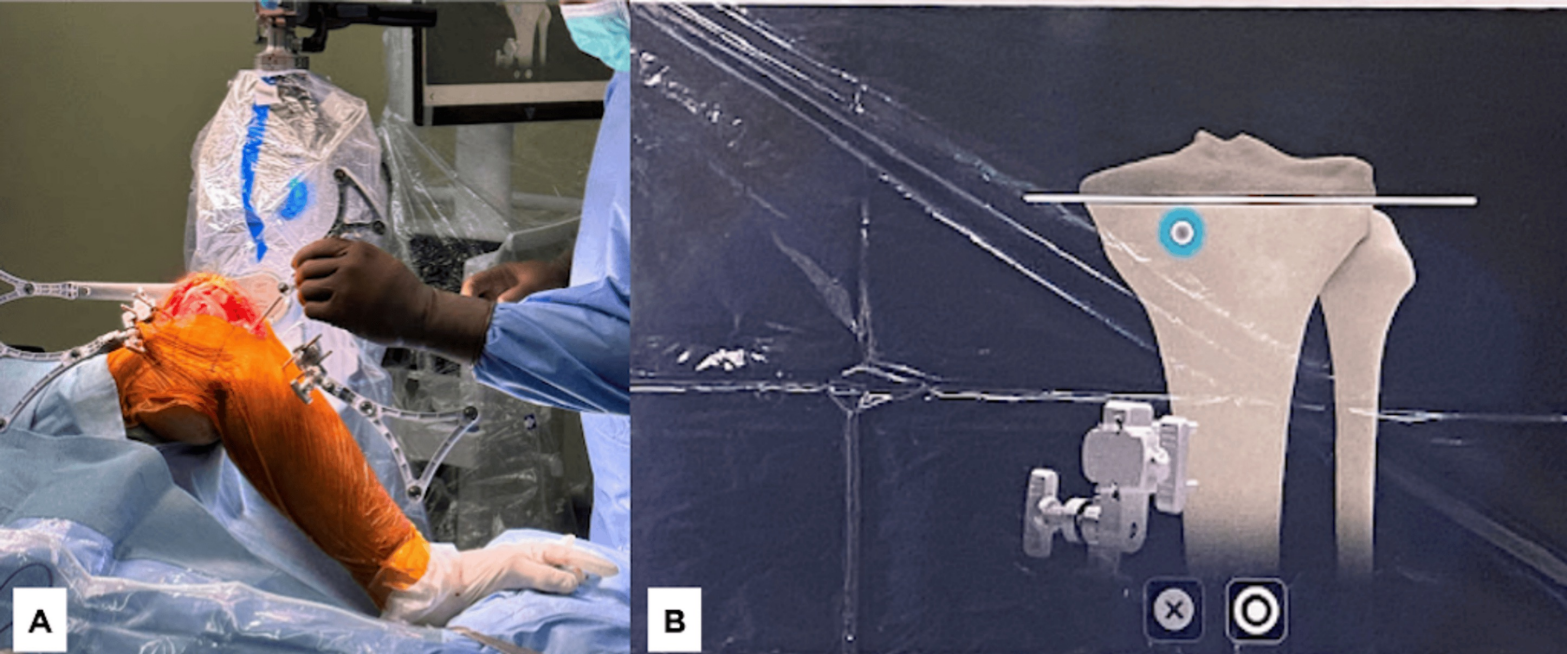
(A) The surgeon points to the lowest medial tibial plateau to measure and verify that the tibia array has remained stable, as prompted on the screen. (B) The monitor displays the anatomical location that must be indicated by the surgeon using a pointer.

Bone checkpoints are crucial during surgery to guarantee accurate registration and alignment. These steps will access anatomical landmarks, allowing the system to analyze the joint plane, implant position, soft tissue balance, and alignment for surgical planning. Those bony checkpoints and landmarks were the femoral head center, femoral knee center, tibial knee center, tibial plateau, tibial sagittal axis, femoral component rotation reference, trans-epicondylar axis (optional), distal femoral condyles, posterior femoral condyles, and anterior cortex. Ensure the pointer tip is fully seated at the bottom of the checkpoint by ensuring that the checkpoint indentation is free of debris. Position the pointer approximately perpendicular to the bone surface. To verify that the tibia array has not shifted since the landmark acquisition, place the tip of the pointer at the tibia checkpoint (in this case, the tibial plateau). The system will display the distance between these measured and previously registered points (Figure 4). If the displayed distance differs from the original distance between the pointer tip and the checkpoint, it indicates that the tibia array may have moved and further confirmation may be needed before proceeding.

Initial leg alignment and ACCUBALANCE™ (Johnson & Johnson MedTech, New Brunswick, NJ, USA) graph recording

The VELYS system requires an assessment of the initial range of motion (ROM) and alignment to record the desired final alignment and balance of the knee. The knee is fully flexed and extended with valgus to determine the collectability of the medial side of the knee, and varus stress is given to assess the stability and correctability of the lateral side of the knee. The knee ROM and expected gap are recorded with a graphic monitor showing the balance consequences of mechanical alignment. In the initial surgical planning, the graph will show the calculated gaps between the femoral implant and the tibial resection (or tibial insert).

Surgical planning

**Figure 5:**
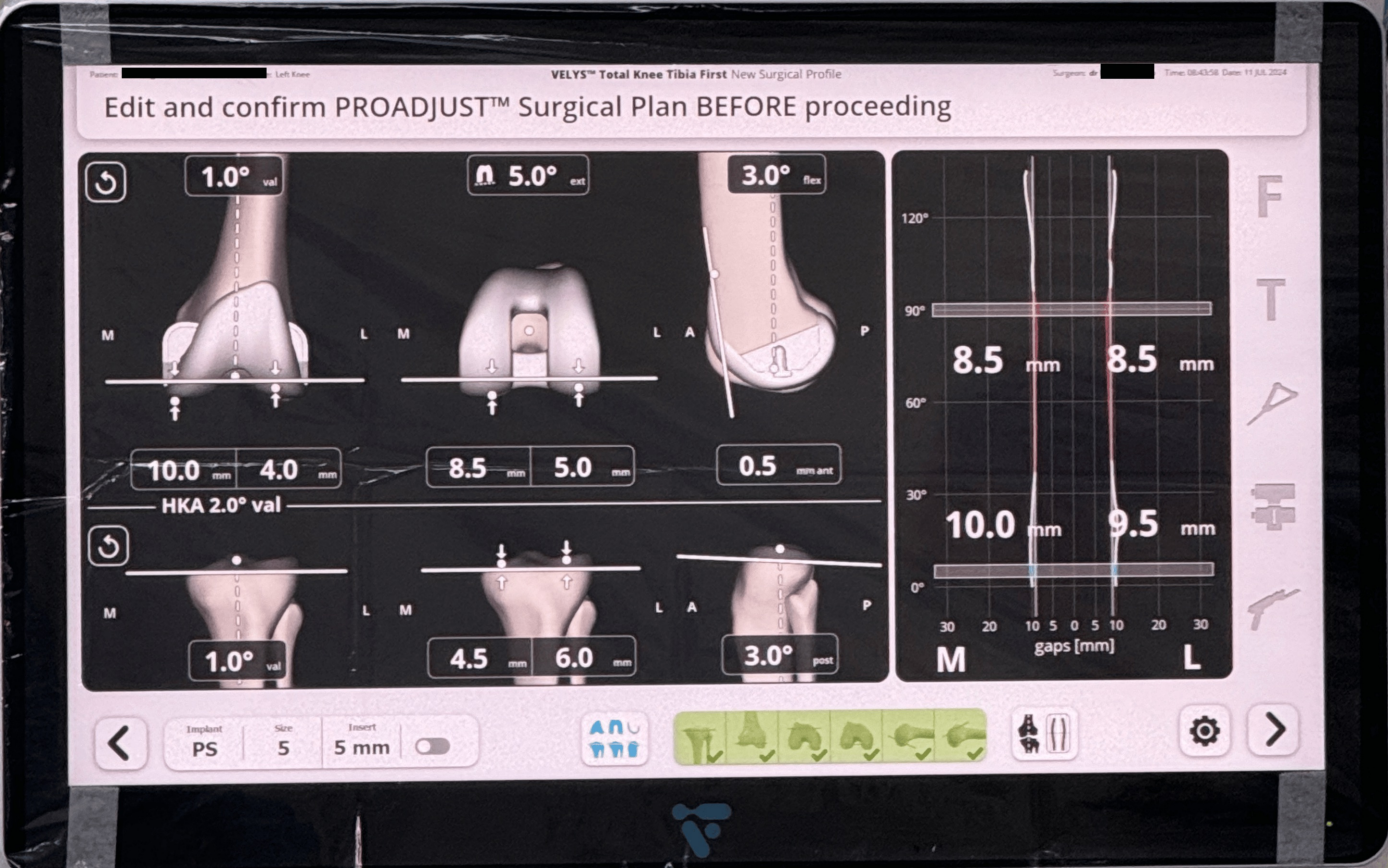
The screen displays the initial surgical planning showing the anatomical placement of the TKR in flexion and extension. TKR: total knee replacement

The goal of this PROADJUST™ surgical plan (Johnson & Johnson MedTech, New Brunswick, NJ, USA) is to accurately plan the size and position of the implant by adjusting bony resection. The initial implant size is determined automatically based on the landmark and graph (Figure 5). The surgeon can change the gap balancing based on the value they find acceptable. 

Tibial resection

**Figure 6:**
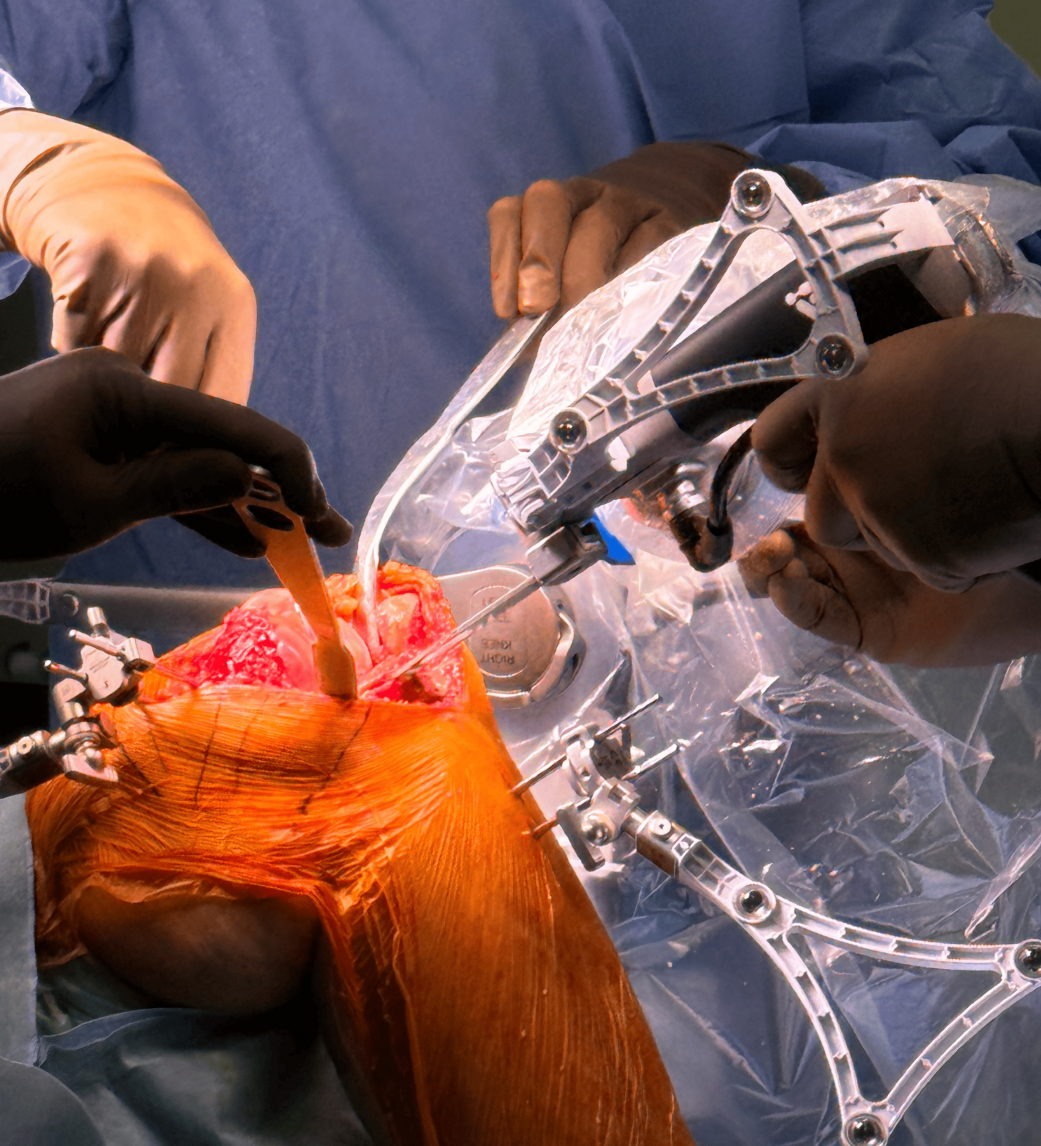
Tibial resection using the robotic arm while the surgeon presses the trigger to start the resection.

The pointer tip is placed one more at the tibia checkpoint to ensure the tibia array has not moved since the landmark acquisition before resection. The resection device is moved until it matches the location shown on the monitor. It will then move on its own to the resection plane. The tibia array must always be visible to the camera during the resection. The leg must be stabilized because any leg movement may lead to inaccuracy (Figure 6).

After the tibial resection, remove the debris from the tibial surface, hold, and place the pointer on the surface to compare the resected and planned resection plane. The robotic device will then move back to its initial position. 

**Figure 7:**
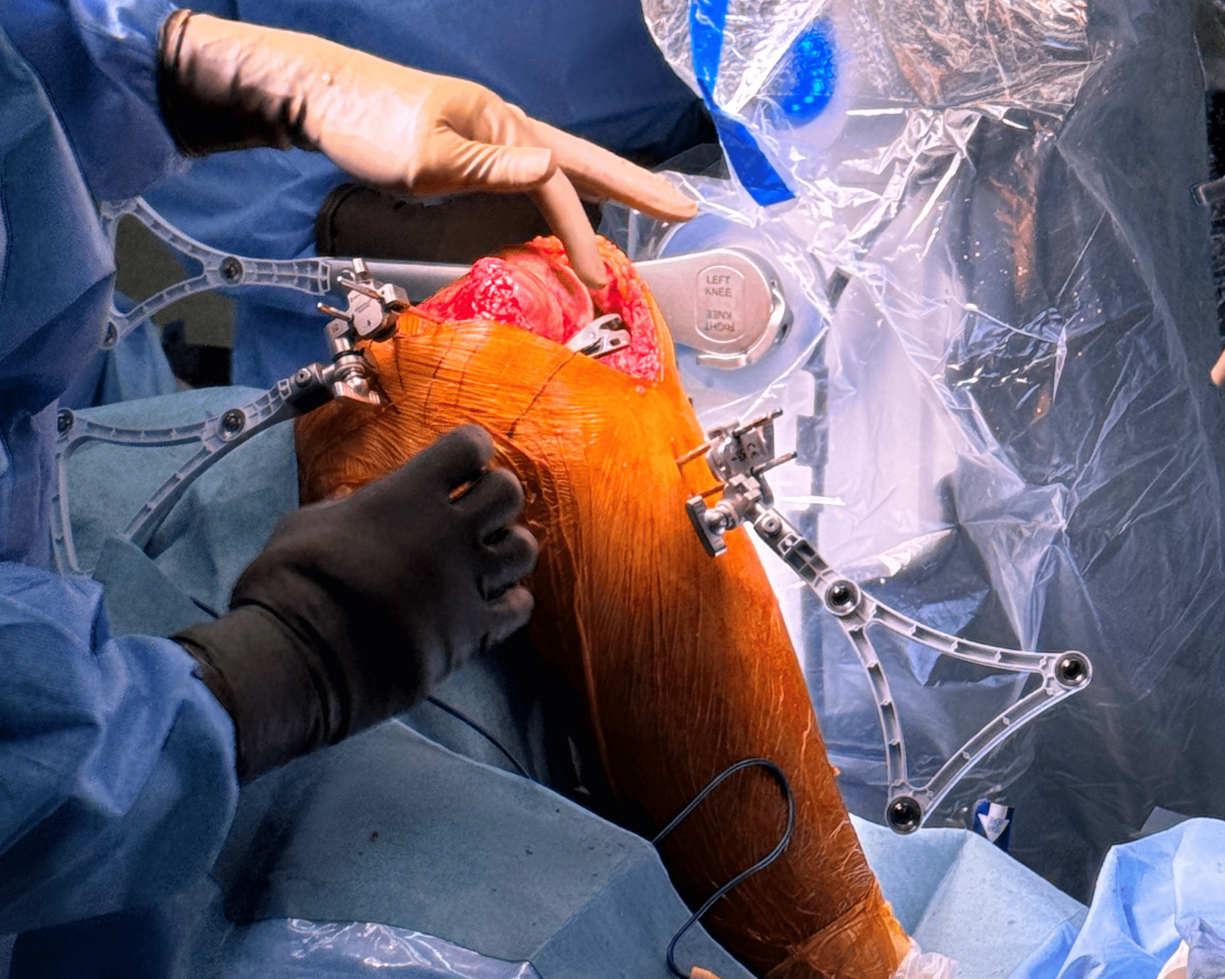
The ligament tensor is inserted.

The ligament tensor is inserted, and the knee is moved to its full ROM (Figure 7). In the varus position, the knee will tighten on the lateral side, while giving the valgus force will tighten the medial collateral ligament. The ACCUBALANCE™will update a new calculated graph. 

Femoral resection

The femoral component can be adjusted before doing a femur checkpoint. Femur checkpoint must be done again before the resection to ensure the femoral location and resection planning. Similar to the tibial resection, the pointer tip has to be moved and point to the exact location until it matches the anatomical location shown on the monitor. 

**Figure 8:**
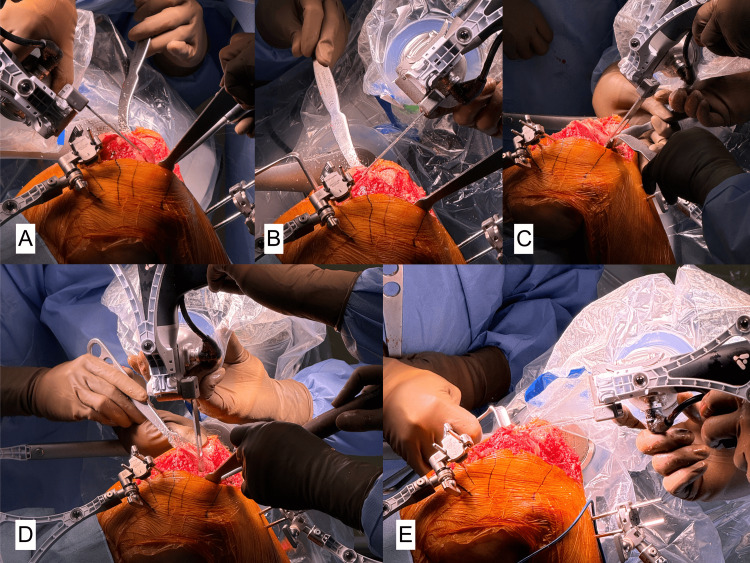
(A) Resection of the distal femur. (B) Resection of the anterior femur. (C) Resection of the posterior femur. (D) Resection of the posterior chamfer. (E) Resection of the anterior chamfer.

The robotic-assisted device will autonomously move to the resection plane, and the tibia array must remain visible to the camera at all times. There are five parts for femoral resection: distal femur, anterior femur, posterior femur, posterior chamfer, and anterior chamfer (Figure 8). From each step, the monitor will guide what part is being resected.

After the resection is completed, the robotic device will return to its initial "home" position and can be moved away from the operating field.

Trial implant, leg alignment, and ACCUBALANCE™ graph

**Figure 9:**
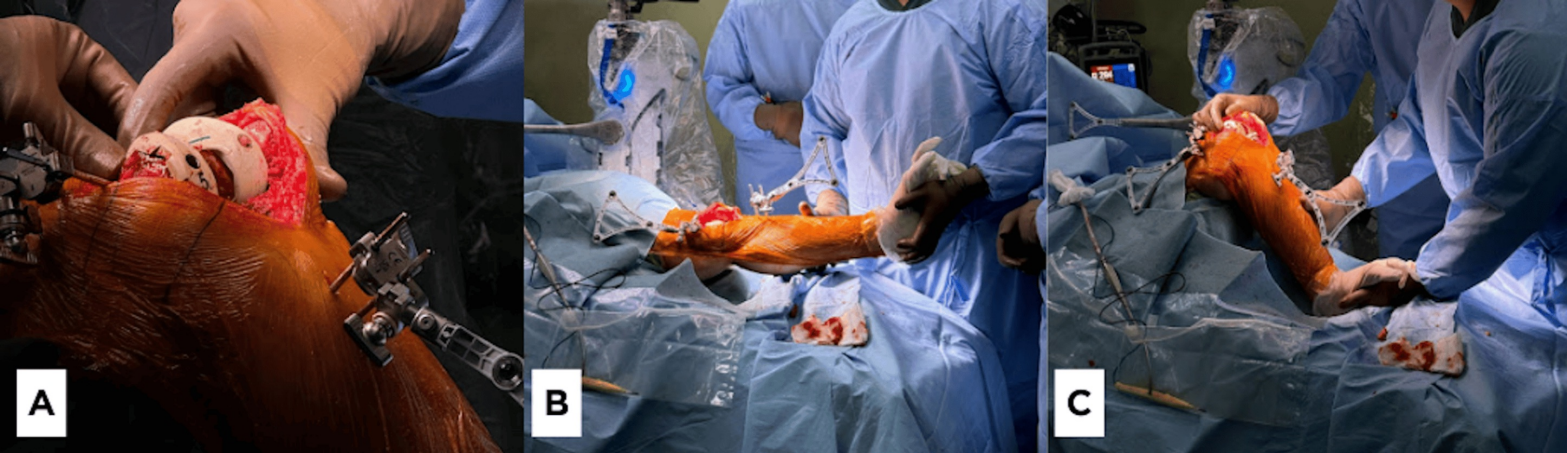
(A) The trial implant is inserted. (B) The knee is fully extended with the trial implant. (C) The knee is fully flexed with the trial implant.

**Figure 10:**
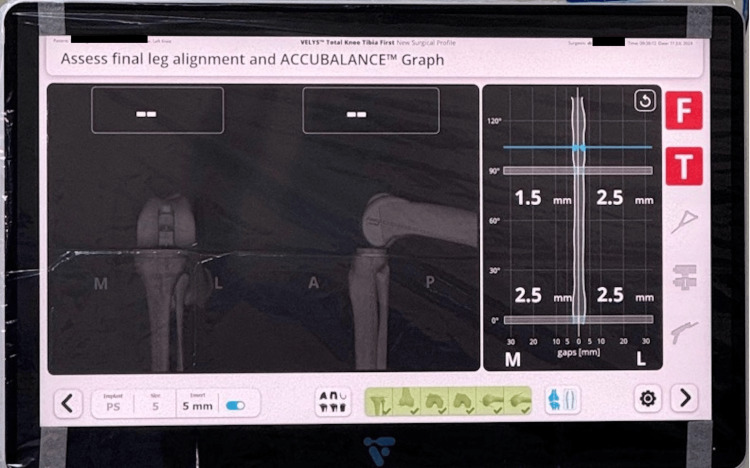
Graph recorded by ACCUBALANCE™ to measure the gap between the femur and tibia when the trial implant is inserted.

The trial implant was then inserted to confirm the knee was well-balanced and well-aligned, and a new ACCUBALANCE™ graph was recorded. The knee was then extended and flexed to assess the final knee ROM. In extension, valgus and varus stress are given to the knee (Figure 9). The graph will display the gap measured between the femoral implant and the planned surface of the tibial insert (Figure 10). Negative numbers (red line) mean a measured gap between the planed femoral implant and tibial resection is smaller than the tibial construct (tibial plateau and insert thickness selected).

Final implant

**Figure 11:**
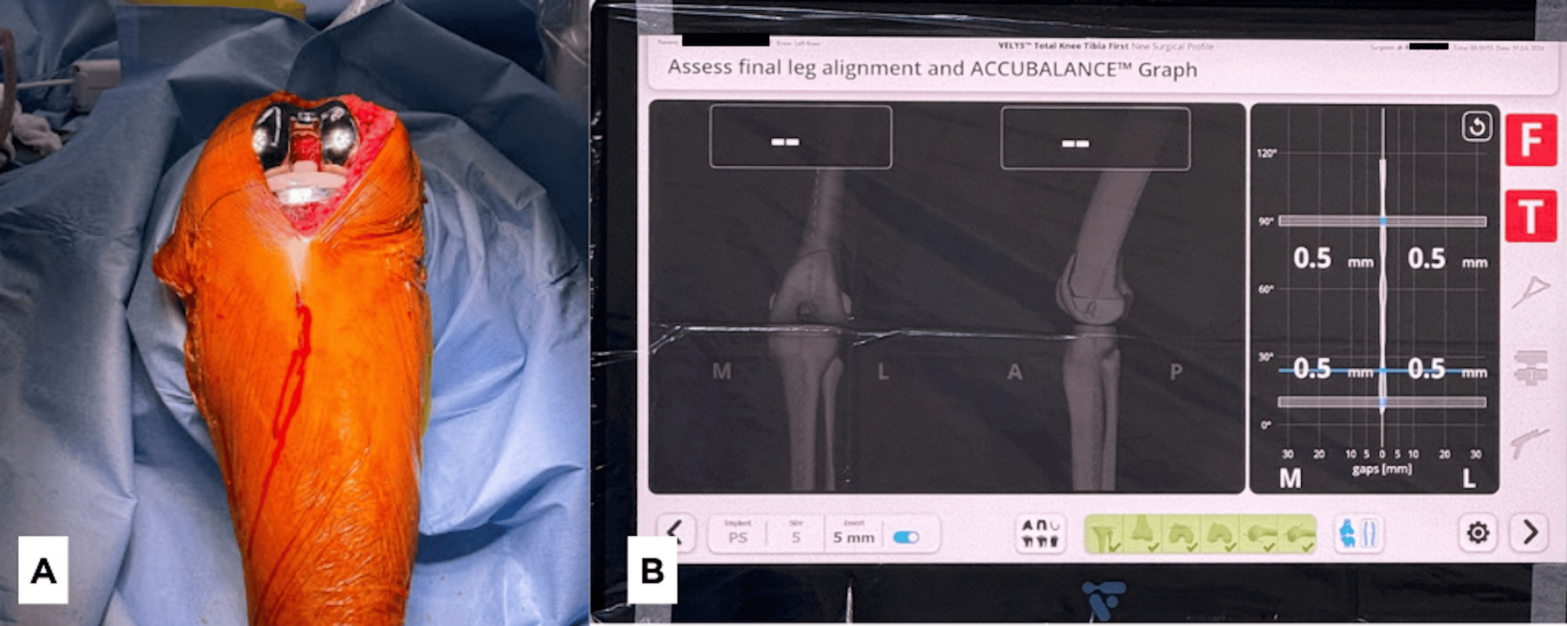
(A) The final implant inserted into the knee joint. (B) Graph recorded by ACCUBALANCE™ with the final implant.

The final implant is inserted, and ACCUBALANCE™ can be recorded once again to measure the final alignment and gap (Figure 11).

## Discussion

Accuracy

Robotic-arm-assisted TKR utilizes bone registration to ensure accurate intraoperative spatial orientation of the limb, with fixed arrays precisely tracking the femoral and tibial bone resection areas throughout the procedure. Stereotactic boundaries limit bone resection to the defined haptic windows, minimizing manual errors and reducing the risk of iatrogenic soft tissue injury that can occur with the handheld sawblade used in traditional TKR, which will ultimately increase the accuracy rate [5]. Accuracy rate can be measured using radiograph, mechanical alignment, or posterior condylar offset ratio (PCOR). Song et al. conducted a randomized study of 100 participants who underwent TKR. They found that the robotic-assisted TKR using the ROBODOC® system group (Curexo Technology Company, Fremont, CA, USA) achieved better accuracy in mechanical alignment and reduced more than 3° outliers [6]. A cadaveric study by Singh et al. found that resection and implant alignment errors in robotic-assisted TKR are less or equivalent to those in conventional methods [7]. A similar study reported that robotic-arm-assisted TKR demonstrated greater accuracy and precision in bone cuts and implant placement compared to conventional methods [8].

Based on the Knee Society roentgenographic evaluation system, the alignment of the femoral and tibial components was assessed using four distinct angles measured on anteroposterior (AP) and lateral radiographs of the examined knees: α (on the femoral coronal plane, the angle between the femoral anatomical axis and the joint line), β (tibial coronal inclination), γ (the femoral sagittal inclination), and δ (tibial sagittal inclination) [9]. A previous study by Kim et al. stated the importance of proper prosthetic implantation alignment and the posterior inclination of the tibial component affects postoperative ROM and prosthetic stability [10]. The result of a systematic review and meta-analysis by Ren et al. with seven studies concluded patients who underwent robotic-assisted TKR show lower deviation values of β, γ, and δ angles and the rate of mechanical outlier is lower in the robotic-assisted group; therefore, robotic-assisted TKR enhances implant accuracy and also reduced alignment errors in sagittal and coronal planes [11].

Inadequate correction of soft tissue imbalances, unbalanced flexion-extension gaps, and knee instability following primary TKR are significant factors contributing to early TKR failures [12,13]. The balanced knee has been associated with better clinical outcomes and satisfaction than the unbalanced knee [14]. Bone cuts in robotic-assisted TKR are more precise and accurate, and the surgeon can potentially achieve gap balance in the knee more precisely [14]. In contrast, a recent study reported asymmetric gap balancing during kinematic alignment TKR with robotic assistance. Patients who experienced a slight increase in lateral laxity, both in extension and in flexion, demonstrated knee kinematics closer to normal [15].

Knee function and ROM

A systematic review conducted by Onggo et al. included 18 studies showing a clinically significant difference in the Hospital for Special Surgery Knee score between robotic and conventional TKR, with the robotic-assisted group having a better score [16]. Clatworthy compared two prospective studies of TKR using robotic and navigation systems. The result shows that the robotic-assisted TKR group shows a better Forgotten Joint score and Western Ontario and McMaster Universities Osteoarthritis Index (WOMAC) score [4].

Fary et al. observed that the active ROM was significantly greater in patients who underwent robotic-assisted TKR than in the conventional method. At one-month follow-up, the ROM improvements were 5.1°, and in three months, they were 2.9° greater in the robotic-assisted group than in the conventional group [17]. A precise bone cut may cause it to be performed in the robotic-assisted group, which may decrease the soft tissue injury and inflammatory markers [5,17]. Other than that, robotic-assisted implants allow for more patient-specific implant placement [18].

A systematic review and a meta-analysis conducted by Ren et al. found no significant difference in knee ROM in patients who underwent robotic-assisted and conventional methods in six months and two years of follow-up [11].

Future directions

With the progression of technology, robotic-assisted TKR is now being used worldwide. Robotic assistance reduces complications and automates repetitive tasks prone to alterations and human errors. Additionally, it is anticipated to improve surgical precision, personalization, and patient outcomes. Furthermore, this technology may be particularly beneficial for more complex cases.

## Conclusions

The VELYS device offers exceptional precision in recording both bony anatomy and soft tissue. These techniques enhance surgical accuracy by enabling precise bone registration and resection, reducing manual errors, and minimizing soft tissue injury compared to conventional methods. They also improve knee gap balancing and reduce the risk of early TKR failure. However, the long-term benefits still require further investigation.

## References

[1] Ben-Shlomo Y, Blom A, Boulton C (2020). The National Joint Registry 17th Annual Report 2020. National Joint Registry - 17th Annual Report.

[2] Prakash R, Agrawal Y (2023). Robotic technology in total knee arthroplasty. Br J Hosp Med (Lond).

[3] Lustig S, Sappey-Marinier E, Fary C, Servien E, Parratte S, Batailler C (2021). Personalized alignment in total knee arthroplasty: current concepts. SICOT J.

[4] Clatworthy M (2022). Patient-specific TKA with the VELYS™ robotic-assisted solution. Surg Technol Int.

[5] Kayani B, Konan S, Pietrzak JR, Haddad FS (2018). Iatrogenic bone and soft tissue trauma in robotic-arm assisted total knee arthroplasty compared with conventional jig-based total knee arthroplasty: a prospective cohort study and validation of a new classification system. J Arthroplasty.

[6] Song EK, Seon JK, Yim JH, Netravali NA, Bargar WL (2013). Robotic-assisted TKA reduces postoperative alignment outliers and improves gap balance compared to conventional TKA. Clin Orthop Relat Res.

[7] Singh V, Teo GM, Long WJ (2021). Versatility and accuracy of a novel image-free robotic-assisted system for total knee arthroplasty. Arch Orthop Trauma Surg.

[8] Hampp EL, Chughtai M, Scholl LY, Sodhi N, Bhowmik-Stoker M, Jacofsky DJ, Mont MA (2019). Robotic-arm assisted total knee arthroplasty demonstrated greater accuracy and precision to plan compared with manual techniques. J Knee Surg.

[9] Ewald FC (1989). The Knee Society total knee arthroplasty roentgenographic evaluation and scoring system. Clin Orthop Relat Res.

[10] Kim YH, Park JW, Kim JS, Park SD (2014). The relationship between the survival of total knee arthroplasty and postoperative coronal, sagittal and rotational alignment of knee prosthesis. Int Orthop.

[11] Ren Y, Cao S, Wu J, Weng X, Feng B (2019). Efficacy and reliability of active robotic-assisted total knee arthroplasty compared with conventional total knee arthroplasty: a systematic review and meta-analysis. Postgrad Med J.

[12] Faschingbauer M, Reichel H (2021). Revision TKA due to instability: diagnostics, treatment options and outcomes [Article in German]. Orthopade.

[13] Hannon CP, Kruckeberg BM, Pagnano MW, Berry DJ, Hanssen AD, Abdel MP (2022). Revision total knee arthroplasty for flexion instability: a concise follow-up of a previous report. Bone Joint J.

[14] Golladay GJ, Bradbury TL, Gordon AC (2019). Are patients more satisfied with a balanced total knee arthroplasty?. J Arthroplasty.

[15] Valpiana P, Salvi AG, Ghirardelli S (2024). Asymmetric gap balancing improves knee kinematic following primary total knee arthroplasty. Arthroplasty.

[16] Onggo JR, Onggo JD, De Steiger R, Hau R (2020). Robotic-assisted total knee arthroplasty is comparable to conventional total knee arthroplasty: a meta-analysis and systematic review. Arch Orthop Trauma Surg.

[17] Fary C, Cholewa J, Ren AN, Abshagen S, Anderson MB, Tripuraneni K (2023). Multicenter, prospective cohort study: immediate postoperative gains in active range of motion following robotic-assisted total knee replacement compared to a propensity-matched control using manual instrumentation. Arthroplasty.

[18] Yamamoto A, Kaneko T, Takada K, Yoshizawa S (2023). Robotic-assisted total knee arthroplasty improves the rotational mismatch between femoral and tibial components, but not the forgotten joint score 12: a single-center retrospective cohort study. J Exp Orthop.

